# Impact of Cadmium on Intracellular Zinc Levels in HepG2 Cells: Quantitative Evaluations and Molecular Effects

**DOI:** 10.1155/2015/949514

**Published:** 2015-08-03

**Authors:** Chiara Urani, Pasquale Melchioretto, Maurizio Bruschi, Marco Fabbri, Maria Grazia Sacco, Laura Gribaldo

**Affiliations:** ^1^Department of Earth and Environmental Sciences, University of Milan Bicocca, Piazza della Scienza 1, 20126 Milan, Italy; ^2^Department of Clinical and Experimental Medicine, University of Insubria, 21100 Varese, Italy; ^3^Molecular Biology and Genomics Unit, Institute for Health and Consumer Protection, DG JRC, European Commission, Via Enrico Fermi 2749, 21027 Ispra, Italy; ^4^Chemical Assessment and Testing Unit (CAT), Institute for Health and Consumer Protection, DG JRC, European Commission, Via Enrico Fermi 2749, 21027 Ispra, Italy

## Abstract

Cadmium is classified as a human carcinogen, and its disturbance in zinc homeostasis has been well established. However, its extent as well as molecular mechanisms involved in cadmium carcinogenesis has yet to be fully clarified. To this end, we used the zinc specific probe Zinquin to visualize and to quantitatively evaluate changes in the concentration of labile zinc, in an *in vitro* model of human hepatic cells (HepG2) exposed to cadmium. A very large increase (+93%) of intracellular labile zinc, displaced by cadmium from the zinc proteome, was measured when HepG2 were exposed to 10 *µ*M cadmium for 24 hrs. Microarray expression profiling showed that in cells, featuring an increase of labile zinc after cadmium exposure, one of the top regulated genes is *Snail1* (+3.6), which is included in the adherens junction pathway and linked to cancer. In the same pathway *MET*, *TGF-βR*, and two members of the Rho-family GTPase, *Rac*, and *cdc42* all implicated in the loss of adherence features and acquisition of migratory and cancer properties were regulated, as well. The microRNAs analysis showed a downregulation of miR-34a and miR-200a, both implicated in the epithelial-mesenchymal transition. These microRNAs results support the role played by zinc in affecting gene expression at the posttranscriptional level.

## 1. Introduction

Cadmium is a highly persistent pollutant harmful to humans and animals, listed as one of the top ten hazardous substances by the Agency for Toxic Substances and Disease Registry [1], and classified as a human carcinogen, based on epidemiological studies and animal experiments, by the International Agency for Research on Cancer [2]. The widespread presence of Cd^2+^ makes it a severe environmental health problem that needs to be considered thoroughly. Apart from natural sources, major fonts of exposure are cadmium-contaminated food and water, providing around 30 *μ*g per day for adults, cigarette smoke [3], and cosmetic products [4].

Cadmium enters the cells by utilizing transport pathways typically evolved for essential metals. Varieties of pathways have been suggested and recently reviewed [5]. In mammalian cells well established pathways involve receptor-mediated endocytosis (megalin/cubilin) of Cd-metallothionein complex and cotransport mechanisms by divalent metal ion transporter 1 (DMT1) and ZIP proteins (ZIP8 and ZIP14A/B). A recently discovered candidate pathway for Cd^2+^ entry into cells is through the T-Type calcium channels Ca_v_3.1 [6]. Due to its molecular mimicry, cadmium follows a Trojan horse strategy leading to interference with essential metals homeostasis. In mammalian cells cadmium toxicity has been closely related to zinc homeostasis. Once inside the cells, cadmium is expected to displace zinc in proteins and enzymes in which zinc has a sulphur-dominated coordination sphere, such as the metallothioneins, the zinc sensor MTF-1, and multiple zinc-finger proteins [3, 7, 8]. However, even though the disturbance of cadmium in zinc homeostasis has already been recognized, its extent has not been a matter of study yet.

In this context, the aim of our work was (1) to demonstrate that the exposure of a human hepatic model system (HepG2 cells) to cadmium leads to an increase of the intracellular pool of labile Zn(II) and (2) to quantitatively measure this increase. To this end, we have used a fluorescent probe (Zinquin) specifically developed for the visualization and measurement of intracellular labile zinc ions [9]. A further aim was to analyze the consequences at molecular level of this increase, as zinc is a recognized second messenger and transcriptional regulator [10, 11].

## 2. Materials and Methods

### 2.1. Cell Cultures and Treatments

Human hepatoblastoma cells (HepG2) represent a useful* in vitro *model for cadmium accumulation and toxicity studies, as previously reported [12–14]. Furthermore HepG2 are, among others, a widely used alternative cell system to primary hepatocytes as they retain, under specific cultivation conditions, many metabolic functions of normal liver cells [15] The HepG2cells were routinely cultured in Opti-MEM medium (Life Technologies, Monza, Italy) supplemented with 10% inactivated fetal bovine serum and 1% antibiotics (streptomycin/penicillin). Cells were kept in incubator at constant 37°C under a humidified 5% CO_2_ atmosphere.

For fluorescence microscopy visualization and spectrofluorimetric measurements the cells were cultured either in complete medium (control) or in complete medium containing CdCl_2_ (Cd, 0.1 and 10 *μ*M) or ZnSO_4_ (Zn, 10, 50, and 170 *μ*M) for 24 hrs.

The viability of HepG2 cells exposed to the highest CdCl_2_ (Sigma-Aldrich, Milan, Italy) concentration (10 *μ*M) for 24 hrs was between 80 and 90% and above 80% in ZnSO_4_ (Sigma-Aldrich, Milan, Italy) treated cells, as previously published [16, 17], and confirmed herewith (data not shown).

### 2.2. Fluorescent Visualization of Free Zinc

Cells were seeded on sterile glass coverslips in 35 mm culture plates (80.000 cells/plate) in complete culture medium. The medium was removed 24 hrs after seeding and substituted with Zn- or Cd-containing medium at concentrations indicated in* par. 2.1*. The cells were left with the control or treatment media in incubator for further 24 hrs.

At the end of treatment time, the cells were processed for fluorescent experiments with Zinquin according to Sarwar Nasir et al. [21] with some modifications.

Zinquin [ethyl (2-methyl-8-*p*-toluenesulphonamido-6-quinolyloxy)acetate] is a fluorescent probe used to detect labile intracellular Zn and to reveal Zn in cultured cells. Zinquin is permeable and is readily taken up by the cells and is essentially nonfluorescent until it complexes Zn with a high selectivity [9, 22], thus providing a means for labile zinc detection and visualization. At the end of all treatments, the medium was removed and the cells were rinsed twice with warm PBS and fixed in formaldehyde (3.7% in PBS) for 30 min at 37°C. After rinsing with PBS, the cells were incubated with Zinquin to a final concentration of 25 *μ*M in PBS and left in the dark for 30 min at 37°C for binding with zinc. Samples incubated with PBS alone were used for autofluorescence evaluations of HepG2 cells. The solution was removed and the coverslips were washed twice with PBS and once with distilled water. The dried coverslips were subsequently mounted on glass slides using glycerol and PBS (9 : 1), containing 2.5% (v/v) 1,4-diazabicyclo[2.2.2]octane solution (DABCO) to reduce fluorescence quenching.

Slides were examined with a fluorescent microscope (Zeiss Axioplan) utilizing a standard UV filter set. All images were acquired by a digital camera (CoolSnap-ProColors Media Cybernetics, Bethesda, MA, USA) and stored by Image Proplus software (Media Cybernetics).

### 2.3. Spectrofluorimetry

Spectrofluorimetry experiments were performed essentially according to Coyle and coworkers [22] with minor modifications. HepG2 cells were seeded in 165 cm^2^ flasks (3 × 10^6^ cells/flasks) and left to recover for 24 hrs before treatments with zinc or cadmium, as described in* par. 2.1*. Treated and control cells were harvested by tripsinization, and a small aliquot from the cell suspension was counted by a Coulter counter to have an estimate of cells number in each sample. All cell suspensions were centrifuged (200 ×g, 10 min) and the pellets resuspended in 2 mL of Hanks Balanced Salt Solution (HBSS: 137 mM NaCl, 5.4 mM KCl, 0.3 mM KH_2_PO_4_, 0.33 mM Na_2_HPO_4_, 1.23 mM CaCl_2_ × 2H_2_O, 0.81 mM MgSO_4_ × 7H_2_O, 4.2 mM NaHCO_3_, and 5.6 mM D-Glucose). The cells in suspension were incubated with Zinquin (final concentration 25 *μ*M) in HBSS for 40 min at 37°C. Then the cells were washed three times with HBSS, and fluorescence was measured at an excitation wavelength of 370 nm and emission wavelength of 490 nm (slit width 5–10 nm) in a Jasco FP-777 spectrofluorimeter. Fluorescence intensities are expressed as arbitrary units and are normalized on a fixed number of cells (100.000 cells).

### 2.4. Microarray Expression Profiling

In this study we reanalyze the gene expression data that we recently published [23] and submitted to NCBI repository (accession number GSE3128).

The whole gene expression of HepG2 cells treated with 10 *μ*M Cd was analyzed by Agilent microarray. In order to detect expression changes between treated and control cells the moderated *t* test was applied. Moderated *t* statistics were generated by Limma Bioconductor package. Modulated genes were chosen as those with log⁡2 fold change greater than 2 and a false discovery rate (Benjamini and Hochberg's method) corrected *P* value smaller than 0.05 [24]. All of the above computations were conducted using the *R* statistics programming environment.

A validation of ten genes regulated by 10 *μ*M Cd was done by a real-time quantitative PCR (qPCR) analysis on the same RNA samples that were used for the microarray. The trends of all validated genes were confirmed as presented in our recent paper [23].

The regulated genes have been mapped on the KEGG (Kyoto Encyclopedia of Genes and Genomes) PATHWAY, a collection of manually drawn pathway maps representing molecular interactions and reaction networks [25].

The heat map for visualization of expression profiles was performed with TM4 system for microarray data analysis. In the map, red and green colours means higher and lower expression levels [26].

### 2.5. MicroRNAs Expression Profiling

RNA was extracted using the MIRVANA kit (AMBION) and was reverse transcribed with Taqman MicroRNA Reverse Transcription Kit using Megaplex RT Primers (Applied Biosystems).

MicroRNA expression was measured and quantified using microfluidic cards (Taqman ArrayMicroRNA Cards, set A, V2.2 and set B, V3, Applied Biosystems) allowing the detection of about 754 unique assays specific and four candidate endogenous control assays (Applied Biosystems, Foster City, CA). Samples of control and 10 *μ*M Cd were tested. Analyses were carried out on the ABI Prism 7900HT Sequence Detection System (SDS) (Applied Biosystems). MicroRNA levels were normalized to endogenous control U6 (Applied Biosystems). MiRNAs with a threshold cycle <33 that showed a log⁡2 fold change greater than two in samples treated with cadmium as compared to control samples were considered as induced.

### 2.6. Statistical Analysis

All experiments were performed in triplicate, and two or three different technical replicates were analyzed in each experiment. Data are expressed as mean ± SD. Student's* t*-test was used for sample comparison, and the software package Statgraphics Plus version 5.0 was used (Statistical Graphics Corp., Manugistic Inc., Rockville, MD, USA).

## 3. Results and Discussion

### 3.1. Cd Exposure in HepG2 Cells Increases Intracellular Labile Zn: Qualitative Evaluations and Intracellular Distribution

To provide evidence that exposure of HepG2 cells to Cd induces an increase of intracellular zinc, we have used the zinc-specific fluorescent probe Zinquin to visualize by microscopy and to measure by spectrofluorimetry the fluorescence intensities. “Free” zinc concentration (where the term free is referred to as freely available, zinc readily bound to chelating agents) is maintained by a complex buffering system in the picomolar to nanomolar range in cells grown under normal conditions, while the total zinc in these cells is evaluated in the high *μ*M range [27–29]. In fact, the main pool of intracellular zinc is bound to a vast population of metalloproteins, referred to as the zinc proteome of the cells, or its excess is stored in zinc-containing vesicles or organelles [30]. In this respect, it should be noted that Zinquin not only binds free zinc (reaction (1)), but can also access Zn-protein with open coordination sites to form fluorescent ternary adducts (reaction (2)), or chelating Zn^2+^ from the proteome (reaction (3)) [31, 32]:

(1) $$\begin{array}{c} {\text{Zn}}^{2+}+2\text{ZQ}⟶\text{Zn}{\left(\text{ZQ}\right)}_{2} \end{array}$$

(2) $$\begin{array}{c} \text{Zn-protein}+\text{ZQ}⟶\text{ZQ-Zn-protein} \end{array}$$

(3) $$\begin{array}{c} \text{Zn-protein}+2\text{ZQ}⟶\text{Zn}{\left(\text{ZQ}\right)}_{2}+\text{apo-protein} \end{array}$$

However, most of the zinc bound to proteins is unreactive toward Zinquin. The term “labile” will be used in the following to indicate all of the Zn(II) sensed by Zinquin. The displacement of Zn(II) from the Zn-proteome by Cd(II) and its labilization has been recently demonstrated on the isolated proteome extracted by pig kidney LLC-PK1 cells by using Zinquin as a fluorescent probe [8]. Here, we are investigating the effect of Cd exposure on the Zn-homeostasis in intact human cells.

**Figure 1:**
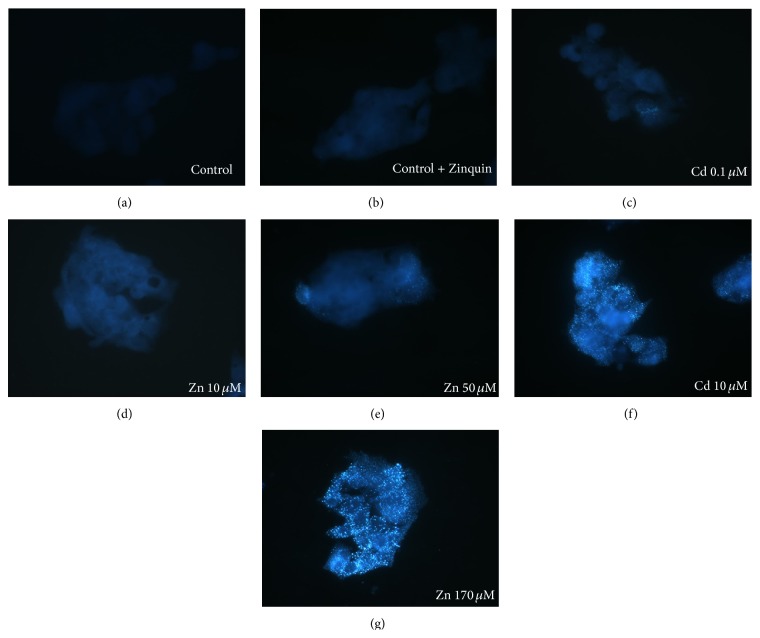
Microscopy images of HepG2 cells loaded with 25 *μ*M of the zinc specific fluorescent probe Zinquin for intracellular visualization and distribution of labile zinc. HepG2 cells grown in control medium without Zinquin were used for autofluorescence visualization (a). Cells grown in control medium show undetectable basal levels of loosely bound zinc (b). HepG2 cells grown in medium containing, respectively, 0.1 (c) and 10 *μ*M (f) Cd for 24 hrs; (d, e, and g): cells exposed to increasing zinc concentrations (10, 50, and 170 *μ*M, resp.). Microscope magnification 400x.

HepG2 cells were exposed to noncytotoxic Cd concentrations (0.1 and 10 *μ*M) and to Zn concentrations representing an overload of the metal (50 and 170 *μ*M). Food and water are major sources of Cd intake for nonoccupationally exposed population. This metal cannot undergo metabolic degradation, is poorly excreted, and is retained in the liver with a very long biological half life (20–30 years) [2], thus providing us with the fact that the maximum concentration used (10 *μ*M) may be compatible with bioaccumulation in the liver. On the other hand, since zinc is an essential element, the cells can buffer high concentrations of this metal. Thus, with the goal of inducing in our cells an observable effect, we exposed HepG2 to high nonphysiological zinc concentrations, up to 170 *μ*M, that was shown not to be cytotoxic [16, 17]. Moreover, the exposure to 10 *μ*M Zn provided the results for a direct comparison to the equimolar concentration of Cd (10 *μ*M). Low physiological labile zinc in the control HepG2 (grown in complete medium) incubated with Zinquin was confirmed as shown by fluorescence microscopy undetectable zinc levels (Figure 1(b)). Cells without Zinquin probing were also analyzed for the evaluation of the background autofluorescence (Figure 1(a)).

The growth of HepG2 cells for 24 hrs in medium containing 10 or 50 *μ*M Zn (Figures 1(d) and 1(e), resp.) or containing 0.1 *μ*M Cd (Figure 1(c)) led to a very low and almost comparable to that of controls or slightly increased fluorescence related to intracellular labile Zn. On the contrary, a dramatic increase in fluorescence intensity was observed when the cells were exposed for 24 hrs to the highest Zn concentration (Figure 1(g), 170 *μ*M Zn), as expected. In these samples, the fluorescence was predominantly distributed in the cytoplasm and in the perinuclear region and had a punctate appearance, as previously reported in other cell types [22, 32]. Notably, an intense and punctuate fluorescence was observed in HepG2 cells grown in culture medium containing 10 *μ*M Cd (Figure 1(f)), showing the high increase of intracellular labile zinc when HepG2 cells are exposed to Cd. These punctuate, vescicular-like structures of high fluorescence intensity were previously designated “zincosomes,” possibly representing late endosomes, at least in certain cells type. These structures may have a role in detoxifying excess zinc increased by toxic agents [10, 30], as in our cells by Cd.

### 3.2. Quantification of Intracellular Labile Zinc in Cd- and Zn-Treated Cells

**Figure 2:**
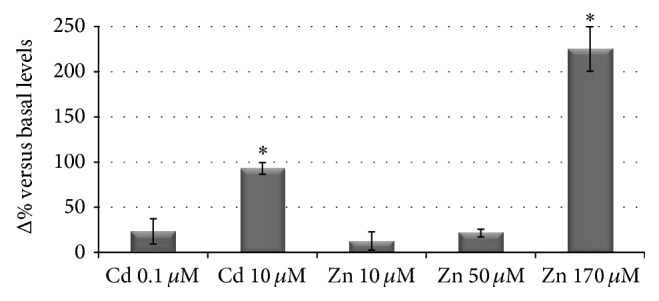
Spectrofluorimetric quantification of intracellular labile zinc. Samples are expressed as Δ% ± SD versus controls (cells grown in control medium in the presence of 25 *μ*M Zinquin). ^∗^ *P* < 0.05 significantly different (Student's *t*-test) from the corresponding Cd or Zn treatment at lower concentration.

The assessment of the amount of intracellular labile Zn in HepG2 cells exposed to excess of Zn or to Cd was carried out by spectrofluorimetric measurements. The fluorescence enhancement after Zinquin binding to intracellular labile Zn was measured as arbitrary units and expressed as a relative increase of the signal with respect to basal control values (cells grown in complete medium). Indeed, it was necessary to express the fluorescence intensities as a Δ% versus controls due to fluctuations of basal conditions possibly due to variations in zinc content amongst different experiments. However, despite this basal difference, the increase was fairly constant within a specific zinc or cadmium treatment, as demonstrated by the relatively small standard deviations obtained in the different biological replicates.  Figure 2 gives an overview of the increment of intracellular labile Zn ions in all treated samples, expressed as fluorescence intensities, while, in Table 1 a summary of all fluorescence intensities expressed as mean ± SD of Δ% of basal fluorescence is presented.

At low zinc concentrations (10 and 50 *μ*M), the intensity of Zinquin fluorescence is relatively low and features mean values of +21% versus basal fluorescence intensities, in agreement with a strict regulation of the cellular zinc content and distribution [10]. Zinquin fluorescence was significantly raised (+225% versus* controls*, *P* < 0.05) in HepG2 grown in the presence of the highest zinc concentration (170 *μ*M), used as a positive control for zinc excess. The most interesting result is the fluorescence intensity of Zinquin bound to intracellular labile Zn in cells grown in the presence of 10 *μ*M Cd: a mean value of +93% versus basal fluorescence intensities was obtained. To the best of our knowledge, this is the first time that the amount of labile zinc is visualized and quantitatively evaluated in Cd-exposed mammalian cells. In our previous works [13, 14] the total intracellular amount of zinc and cadmium in HepG2 cells exposed to 170 *μ*M Zn and 10 *μ*M Cd for 24 hrs was measured by an analytical technique (ICP-AES). The increment in the total concentrations with respect to the basal values was about 0.075 ppm/10^6^ cells for zinc in cells exposed for 24 hrs to 170 *μ*M Zn and 0.050 ppm/10^6^ cells for cadmium in samples exposed to 10 *μ*M Cd. Thus, it can be noted that, at the same exposure time, the uptake of cadmium is only slightly smaller than that of zinc, although cells have grown in the presence of a concentration of cadmium which is about 20 times smaller than that of zinc. This result clearly indicates that the uptake of cadmium, that uses aspecific transport pathways [5], occurs to a significantly larger extent to that of zinc, whose concentration is, on the contrary, strictly regulated. This result is consistent with the large increment of the fluorescence intensity of HepG2 cells exposed to 10 *μ*M Cd as due to cadmium to zinc replacement in proteins and mobilization of the latter metal ion, a mechanism previously proposed [7] and recently demonstrated in a prokaryotic zinc-finger domain [33], and proteome extracted from pig kidney LLC-PK1 cells [8].

### 3.3. Biological Effects of Labile Zinc Increase in HepG2 Cells: Genes and MicroRNAs Regulation

Prompted by the results discussed above, we have reanalyzed the molecular data and pathways to better understand the mechanisms of cadmium biological effects and to correlate the role of zinc in gene and molecular regulation.

**Figure 3:**
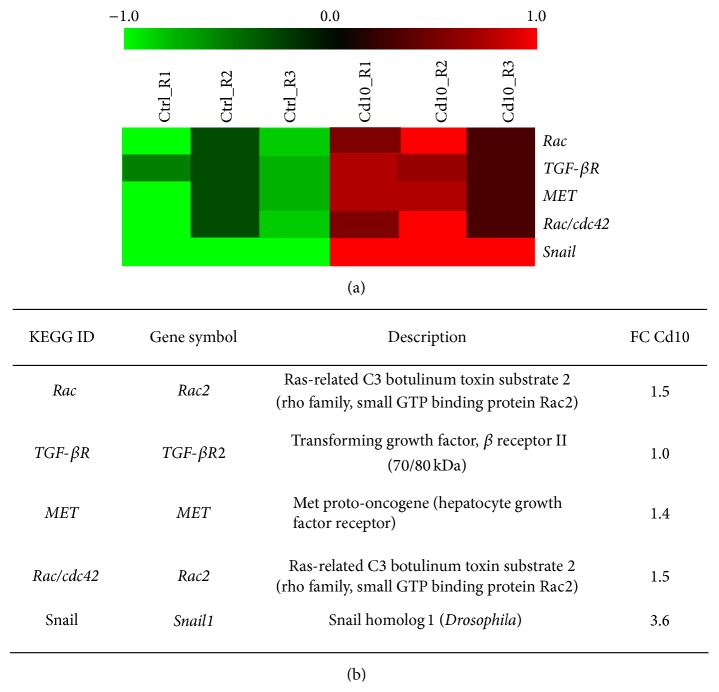
The genes regulated by 10 *μ*M Cd treatment and belonging to adherens junction pathway represented in Figure 4 are displayed. (a) Heat map graphically representing the gene regulation. Each row represents a mRNA (green = low expression, red= high expression). (b) In the table the data are expressed as log⁡2 FC (fold change), comparing HepG2 treated with 10 *μ*M Cd to control cells.

**Figure 4:**
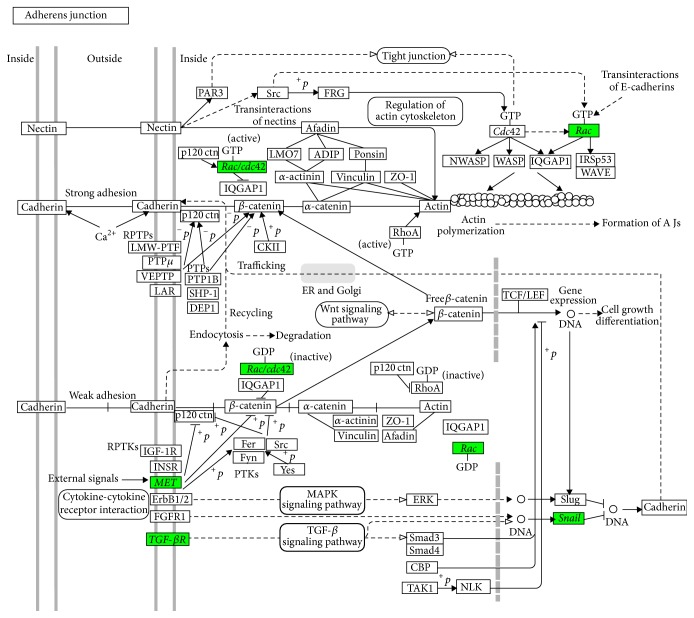
Representation of the adherens junction pathway map from KEGG. Upregulated genes in HepG2 cells by 10 *μ*M Cd treatment for 24 hrs are colored in the signaling pathway map.

The elevated concentrations of Zn(II) visualized and measured in HepG2 cells after 24 hrs of Cd(II) exposure activated a number of zinc-related processes. One of the top regulated genes in our samples, as identified by the Microarray expression profiling (Figure 3), is* Snail1*, with an upregulation of +3.6 fold change with respect to controls. Snail1 is included in many biological pathways, one of which is the adherens junctions, represented in Figure 4. Snail1 belongs to a superfamily of zinc-finger transcription factors involved, among others, in the acquisition of invasive and migratory properties during tumor progression [34]. The mechanism of Snail1 activation has been recently related in breast cancer cells to zinc influx and intracellular zinc increase, either directly or indirectly [35], thus strongly supporting our data.

Other upregulated genes in the adherens junction pathway (Figure 4) are* MET* (1.4 fold change),* TGF-βR* (1 fold change), and the two members of the Rho-family GTPase,* Rac* (1.5 fold change), and* cdc42* (1.5 fold change). The MET tyrosine kinase receptor (known also as the HGF receptor) promotes, among others, cancer growth and metastasis by conveying proliferative, antiapoptotic, and promigratory signals (see for a review [36]). The transforming growth factor receptor (TGF-*β*R) is a member of the TGF-*β* signalling pathway, involved in cell proliferation and cell migration functions. In addition, TGF-*β* is a signalling molecule implicated in* Snail1 *activation and in our samples exposed to Cd is upregulated with a 1.4 fold change [37]. The* Rac* and the* cdc42* genes promote lamellipodia and filopodia formation, thus regulating cell migration through cytoskeleton remodelling (see for a review [38]).

The role of elevated zinc concentrations was demonstrated to affect gene expression in cancer cells at posttranscriptional level [39]. Thus, intriguing results linking zinc levels and gene expression can emerge from microRNAs (miRNA) analysis in HepG2 cells exposed to cadmium. The miRNA are small noncoding RNAs initially transcribed and processed in the nucleus as precursors. These molecules are then exported to the cytoplasm where they become mature miRNA of about 23 nucleotides whose main function is to negatively regulate gene expression at the posttranscriptional level by repression of protein translation or promotion of mRNA degradation [40]. In addition, miRNAs by targeting multiple transcripts play a crucial role in tumorigenesis and cancer progression [41]. Even if the role played by zinc in cell growth and proliferation is well known [10], the involvement of this metal in the regulation of gene expression at posttranscriptional level is still to be explored. Recently it was demonstrated that elevated intracellular zinc concentrations modulate the miRNA expression in mammalian cells, with miR-223 among the top downregulated miRNA [39]. It is important to highlight that miR-223 is involved in cell cycle regulation, proliferation, and survival [42], thus suggesting the relevance of zinc concentrations in the epigenetic mechanisms of cancer, at least those known up to now.

Two major miRNAs were downregulated in our samples: a miR-34 family member (−1.1 fold change) and a miR-200 family member (−1.2 fold change).

Very interestingly, a decrease in miR-34, which normally antagonizes Snail1, was recently described as part of the p53/miRNA-34 axis. Namely, in the absence of a functional p53 and of a decrease of miRNA-34,* Snail1* is upregulated, as we found in our samples. This axis promotes the epithelial-mesenchymal transition (EMT) and the invasion program in neoplastic cells [20]. Further supporting this mechanism are our results in HepG2 cells on miR-200 family. The miR-200 family has been related to the suppression of the epithelial-mesenchymal transition as well. A correlation between downregulation of the miR-200 family member and induction of TGF-*β*1 in renal proximal tubule epithelial cells (NRK-52E) was observed and are responsible for the epithelial-mesenchymal transition [43]. On the other hand, an overexpression of the family member miR-200b was demonstrated to significantly reduce cellular proliferation and inhibition of cell migration and metastasis in gastric carcinoma cells [44]. All these data strengthen the correlation between high zinc levels,* Sanil1* upregulation, miR-34, and miR-200 family members downregulation.

## 4. Conclusions

We have previously demonstrated [23, 45] that Cd exposure in HepG2 cells modulates genes and proteins responsible for metal homeostasis, such as the metallothioneins (MT) and membrane transporters responsible for the elimination of zinc excess (i.e., ZnT-1). However, we posed [45] a question on ZnT-1 regulation by Cd exposure: how does Cd regulate ZnT-1 levels? We proposed a mechanism of activation by elevated labile zinc concentrations possibly due to the replacement of Zn(II) from the Zn-proteome by Cd(II) and its corresponding labilization. As known, both MT and ZnT-1 are activated by the metal transcription factor-1 (MTF-1) that functions as a metal sensor. MTF-1* in vitro *is robustly regulated by zinc but not by cadmium ions, as just published [46],and further confirming that labile zinc displaced by cadmium is the primary mediator in MTF-1 activation. The regulation of MTF-1 by metals, such as Cd, could be a direct consequence of zinc to cadmium displacement from zinc-containing proteins. The Cd-Zn exchange and the possible substitution of Cd in Zn-binding sites were previously described [7, 18]. The results presented here demonstrate the increment of labile zinc levels in the presence of cadmium, supporting the validity of the mechanism proposed above. Further work is underway by our research group to investigate the gene expression and the biological implications of the modulation of these genes, due to the displacement of zinc with cadmium as a function of their concentrations.

**Figure 5:**
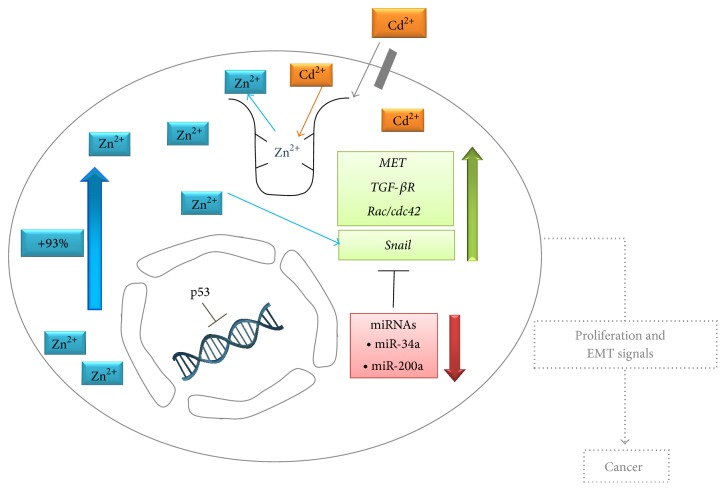
Overview of cellular and molecular effects of 10 *μ*M Cd in HepG2 cells. Cd enters the cells through aspecific sites. Intracellular Cd accumulation determines an increase of labile zinc, a known proliferation signal, and second messenger. The replacement of Zn with Cd in the zinc proteome [18] is the hypothetical process underlying the above described mechanism. Genes (*Snail*,* MET*,* TGF-βR*, and* Rac/cdc42*) involved in the loss of cell adherence and also comprised in the mechanism of epithelial-mesenchymal transition are disregulated. MicroRNAs (miR-34a, miR-200a) with tumor-suppressor functions are downregulated. Cd-exposed HepG2 cells have a nonfunctional p53 [19] that along with miR-34 downregulation represents the disregulated axis in* Snail1*-dependent epithelial-mesenchymal transition [20].

Taking into account our present and previously published data, the following mechanisms emerge (Figure 5): (i) HepG2 cells in the presence of Cd have a nonfunctional p53 (previously published data [19]); (ii) Cd determines an increase of intracellular labile zinc level, which is a known proliferation signal [10]. This response leads to the activation of* Snail1,* that along with the upregulation of other genes responsible for loss of adherence, represents a signal promoting cell migration and metastasis (present work, Figure 4 adherens junction); (iii) miR-34 downregulation contributes to the abnormal expression of* Snail1*, which is normally antagonized by miR-34 and whose pathological expression has been linked to cancer cell epithelial-mesenchymal transition; (iv) miR-200 downregulation contributes to the epithelial-mesenchymal transition as well.

Therefore, our results provide new information on the existing proposed mechanisms for Cd toxicity and biological effects and along with those already reported in the literature open new intriguing interpretations on Cd-induced carcinogenicity.

## Figures and Tables

**Table 1:** 

Cd 0.1 *µ*M	Cd 10 *µ*M	Zn 10 *µ*M	Zn 50 *µ*M	Zn 170 *µ*M
23.17 ± 14.01	93.03 ± 6.55^*^	12.57 ± 10.24	21.37 ± 4.24	225.22 ± 24.59^*^

Fluorescence intensities (arbitrary units) of labile zinc in cadmium- or zinc-treated HepG2 cells. The results are expressed as increment of basal (control) values ± SD of at least three independent biological replicates. ^*^ *P* < 0.05 significantly different (Student's *t*-test) from the corresponding Cd or Zn treatment at lower concentration.

## Highlights

- HepG2 cells exposed to Cd show an increase of intracellular labile zinc.
- Labile zinc is visualized and measured by the zinc specific probe Zinquin.
- Genes belonging to loss of adherence and cell migration pathways are activated.
- Cd exposure affects the expression of miR-34a and miR-200a.
